# Analysis of the discontinuation and nonpublication of neurooncological randomized clinical trials

**DOI:** 10.1093/noajnl/vdae136

**Published:** 2024-08-01

**Authors:** Molly Butler, Mehul Mehra, Abdullah Chandasir, Lydia Kaoutzani, Fernando L Vale

**Affiliations:** Medical College of Georgia at Augusta University, Augusta, Georgia, USA; Medical College of Georgia at Augusta University, Augusta, Georgia, USA; Medical College of Georgia at Augusta University, Augusta, Georgia, USA; Wellstar-Medical College of Georgia Health, Department of Neurosurgery, Augusta, Georgia, USA; Wellstar-Medical College of Georgia Health, Department of Neurosurgery, Augusta, Georgia, USA

**Keywords:** clinical trials, discontinuation, neuro-oncology, nonpublication

## Abstract

**Background:**

Premature discontinuation and nonpublication of clinical trials contribute to research waste and compromise our ability to improve patient outcomes. However, the extent to which these problems exist in neurooncological randomized clinical trials (RCTs) is not known. This study aimed to evaluate the prevalence of discontinuation and nonpublication of neurooncological RCTs, identify contributing factors, and assess trial characteristics associated with each.

**Methods:**

We performed a retrospective, cross-sectional study of neurooncological RCTs registered in Clinicaltrials.gov before March 7, 2023. Data were collected from Clinicaltrials.gov and associated publications were located. We attempted to contact authors for all trials without associated publications or an identified reason for discontinuation.

**Results:**

Of 139 included RCTs, 57 (41%) were discontinued. The most common reason for discontinuation identified was slow enrollment or accrual (23%), though 30 trials (53%) were discontinued for unknown reasons. Trials funded by sources other than industry or the National Institutes of Health were more likely to be discontinued (odds ratio 4.2, 95% confidence interval 1.3–13.8). In total, 67 of the 139 (48%) RCTs were unpublished, including 50 of the 57 (88%) discontinued studies and 17 of the 82 (21%) completed studies.

**Conclusions:**

In our study, discontinuation of neurooncological clinical trials was common and often occurred for unknown reasons. Trials were also frequently unpublished, particularly those that were discontinued. Addressing these findings may provide an opportunity to reduce research waste and improve outcomes for patients with neurological cancers.

**Key Points**

- Nearly half of the neurooncological trials in our study were discontinued.
- Trial discontinuation often occurred due to slow enrollment or for unknown reasons.
- Neurooncological trials were commonly unpublished, especially those that were discontinued.

**Importance of the Study**

Randomized clinical trials (RCTs) are instrumental in driving evidence-based medicine and advancing the standard of care for patients across all medical specialties. Previous studies have shown that the discontinuation and nonpublication of RCTs may limit this advancement and contribute to research waste, but the extent to which these problems exist in neurooncological RCTs has not been well understood. The findings of our study highlight the prevalence of discontinuation and nonpublication in neurooncological RCTs, identify contributing factors, and describe characteristics associated with discontinued and unpublished trials. Our study provides valuable insight for clinicians and researchers conducting clinical trials and may serve as a guide for the implementation of targeted interventions to reduce inefficiencies in neurooncological research.

Clinical trials are crucial for the advancement of evidence-based medicine. However, the conduction of randomized clinical trials (RCTs) often requires extensive financial resources. For instance, a study of trials from 2015 to 2017 estimated a median cost of $41 413 per patient.^1^ Despite this cost and the finite resources allotted for medical research, potentially wasteful practices remain common among clinical trials. Studies have estimated that up to 85% of investment in biomedical research is wasted, representing approximately 200 billion dollars in 2010.^2^ Many factors can contribute to the inefficient use of resources in clinical trials, but notable causes include premature trial discontinuation and failure to adequately report a trial’s findings.^3^

Understandably, clinical trials may be discontinued for reasons related to the safety, efficacy, or feasibility of the study. However, many trials are discontinued for unrelated or preventable reasons.^4^ In addition, it has been recognized that only a fraction of all clinical studies ultimately reach publication in a peer-reviewed journal and approximately half of all clinical trials remain unpublished^.5–7^ Previous studies have identified a lack of time or low priority, results not deemed important enough, and journal rejection as contributions to nonpublication.^8^ Regardless of the cause, nonpublication of clinical trials and discontinuation for preventable reasons result in resource consumption while failing to contribute new knowledge to the scientific and medical communities.

Though nonpublication and discontinuation of clinical trials have been identified as prevalent issues in general research populations, the extent to which these problems exist specifically in neurooncological studies is not known. Central and peripheral nervous system tumors are a major public health burden, with a global incidence of 347 thousand and responsible for 246 thousand deaths in 2019.^9^ In 2010, the total cost of neurological cancers in the United States was 4.47 billion dollars.^10^ Clinical trials are therefore especially important for improving treatment options for these patients.^11^

The prevalence and financial burden of neurological cancer necessitate consideration of research waste in designing and conducting clinical trials and efforts to optimize resource consumption. Exploring factors associated with the discontinuation and nonpublication of neurooncological clinical trials therefore represents an opportunity to further our understanding of this issue and identify potential ways to decrease the inefficient use of research resources.

In this study, our primary objective was to investigate the prevalence and characteristics associated with the discontinuation and nonpublication of neurooncological clinical trials and evaluate the underlying reasons for both.

## Materials and Methods

We performed a retrospective, cross-sectional study of neurooncological RCTs registered in Clinicaltrials.gov, an online database provided by the National Library of Medicine. We developed our methodology based on the process previously described for head and neck cancer studies by Johnson et. al.^12^ This study was not subject to Institutional Review Board oversight as it did not meet the definition of human subject research, outlined in 45 CFR 46.102(d) and (f) of the Department of Health and Human Services Code of Federal Regulations.^13^

Using the advanced search function provided by ClinicalTrials.gov, we used the following keywords to locate clinical trials related to central and peripheral nervous system tumors: “neoplasm of spine,” “carcinoma of spine,” “spine cancer,” “cancer of the spine,” “spine tumor,” “brain cancer,” “neoplasm of brain,” “cancer of the brain,” “carcinoma of brain,” “brain tumor,” “cancer of peripheral nerve,” “neoplasm of peripheral nerve,” “peripheral nerve cancer,” and “peripheral nerve tumor.” This search also yielded additional results based on the automated term-mapping capability of ClinicalTrials.gov which were considered for inclusion. Our initial search was performed on March 7, 2023, and included all RCTs that were registered before this date with ClinicalTrials.gov. Results were compiled and formatted in Microsoft Excel Version 16.71.

**Figure 1.**
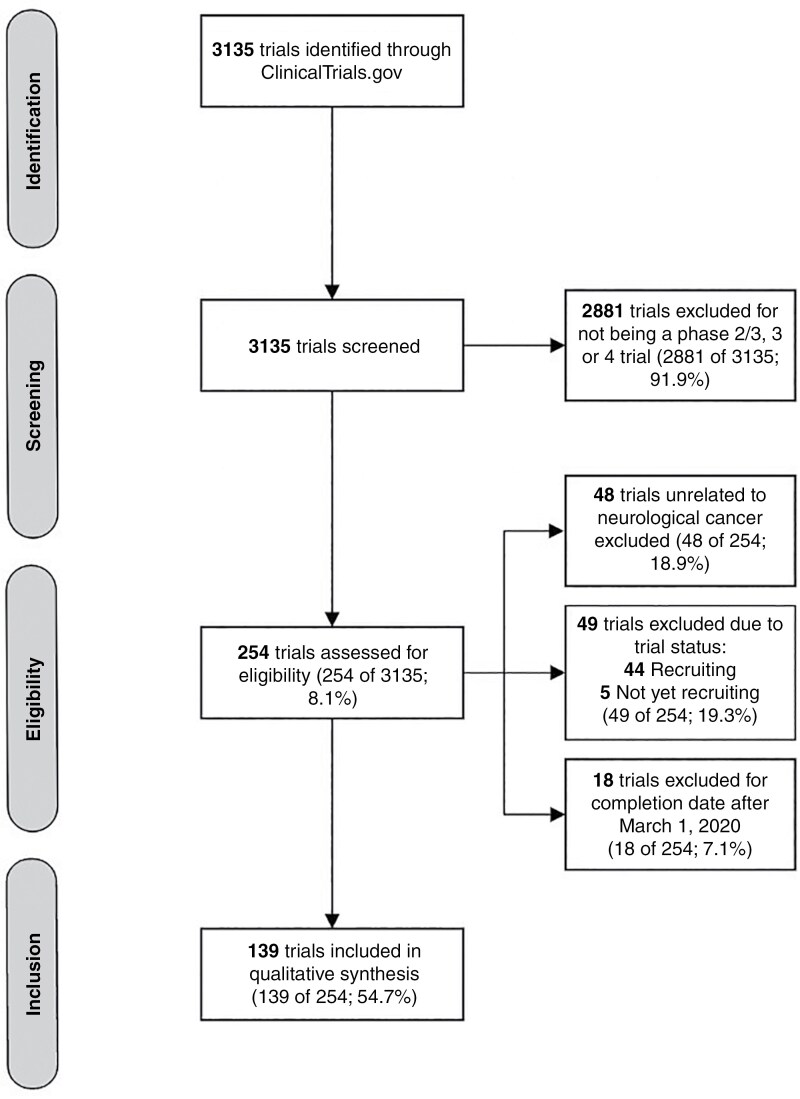
Methodology for selection of included trials.

We then screened all clinical trials from our search by title, condition, study design, and completion date. We excluded trials that were: (1) not related to neurological cancer, (2) not in phase 3 or 4, (3) completed after March 1, 2020, and (4) ongoing or not yet begun (trials with a status of active, recruiting, not yet recruiting, or enrolling by invitation). We chose to focus here on phases 3 and 4 clinical trials because they typically involve a greater number of participants, representing a larger and more diverse patient population, and aim to assess short and long-term outcomes with a stronger impact on clinical decision-making.^14^ Combined phases 2 and 3 trials were also included. Phases 1 and 2 clinical trials were excluded because these trials primarily assess toxicity and safety concerns contributing to a limited impact on clinical practice and are less often published.^15,16^ Clinical trials completed after March 1, 2020 were excluded from the sample to allow investigators 36 months after trial completion for subsequent publication.^17^ However, individual trials that involved participants with non-neurological cancers, in addition to those with neurological cancers, were included in our sample. Figure 1 outlines the strategy we used in selecting trials for inclusion. We then sorted the included clinical trials by trial status. All trials with a status of completed formed the “completed” group, and trials with a status of terminated, withdrawn, unknown, or suspended formed the “discontinued” group. If trials with a status of terminated, withdrawn, unknown, or suspended were later found to be completed via correspondence with the investigator, they were moved to the completed group for further analysis. Similarly, trials with a completed status were moved to the discontinued group for analysis if they were later found to be discontinued.

We reviewed ClinicalTrials.gov to identify the publication status of trials in the completed group. If no publication was linked to the trial’s Clinicaltrials.gov listing, we performed a search of PubMed, Google Scholar, and Embase using the title of the trial, investigator names, and national clinical trial numbers to locate any publications not already identified. If our search process identified trial results in the form of conference abstracts, we included these in our analysis as well. For this study, we considered the publication of clinical trials to include the release of final trial results in a peer-reviewed journal or the presentation of trial results at conferences with corresponding abstracts available online.^18^ If an associated publication for a clinical trial was not found through either of these 2 mechanisms, we attempted to locate an email address for each trial’s corresponding investigator. We first searched Clinicaltrials.gov listings, then searched PubMed and Google Scholar by investigator name to see if an email address could be found associated with his or her other publications. We also performed a Google search of the corresponding investigator and searched the web pages of their respective institutions to locate contact information. If the investigator’s email address was located, we sent a standardized email inquiring about the reason for nonpublication using prespecified responses. The prespecified responses concerning the reason for nonpublication were chosen based on a systematic review by Song et. al and were used with success in a similar study of head and neck cancer clinical trials by Johnson et. al.^12,19^ If we did not receive a response from the author within one week of sending the standardized email, we sent the same email again once per week for 2 additional weeks. We considered an investigator to be uncontactable if we did not receive a response within 8 weeks of sending our initial email or if our email returned as undeliverable. Previous studies have utilized similar methods of correspondence to obtain information from trial investigators.^12,20,21^ For trials with uncontactable corresponding investigators, we repeated the same search process to identify contact information for any other investigator on the trial listing. We sent the same standardized email to these investigators and allowed 2 additional weeks for an email response. We considered a trial to be unpublished if we could not identify an associated publication on Clinicaltrials.gov, through our search process or via correspondence with investigators.

For each clinical trial in the discontinued group, we reviewed Clinicaltrials.gov to identify any listed reasons for the trial’s discontinuation. We also searched through trials’ corresponding publications, if available, to identify any reasons for discontinuation reported there. If a trial’s reason for discontinuation was still unknown, we attempted to contact investigators in the same manner as above. Additionally, for trials with associated publications, we searched for contact information for the first and/or last authors if no email address was identified for investigators on the Clinicaltrials.gov listing.

Summary statistics and logistic regression were both calculated using R version 4.3.2.

Unadjusted and adjusted odds ratio binary logistic regression was used to determine the association between completion and publication status and trial characteristics. This was calculated using R version 4.3.2. Criterion variables in our model were trial status (0 = completed, 1 = discontinued) and publication status (0 = published, 1 = unpublished) with funding source, intervention, and location where trials were conducted as variables.

## Results

### Study Characteristics

The initial search of Clinicaltrials.gov for clinical trials related to neurological cancers yielded 3135 studies. Of these, 2881 were either phase 1 or 2 trials and were excluded. The remaining 254 trials underwent a detailed screening for eligibility. In total, 115 of these 254 trials were deemed ineligible for inclusion: 48 trials were excluded for being unrelated to neurological cancer, 49 trials were excluded for having a trial status of either active, recruiting, not yet recruiting, or enrolling by invitation and 18 trials were excluded for having a completion date after March 1, 2020 (Figure 1). Our final sample was comprised of 139 clinical trials, including 11 combined phases 2 and 3 trials, 100 phase 3 trials, and 28 phase 4 trials. Included trials most investigated pharmacologic interventions (58 of the 139 trials [41.7%]) and a combination of pharmacologic and radiotherapy interventions (26 of the 139 trials [18.7%]). Other interventions are shown in Table 1. Most of the trials were funded by sources other than Industry or the National Institute of Health (NIH; 82 of the 139 trials [59.0%]). Table 1 shows funding sources for the remaining 57 trials. The trial starting dates ranged from April 1992 to December 2021. In our sample, 26 countries were represented in terms of primary trial location. Most trials were conducted in the United States (59 of the 139 trials [42.4%]), followed by China (15 of the 139 trials [10.8%]), and France (7 of the 139 trials [5.0%]).

**Table 1.** Characteristics of Completed Versus Discontinued Trials and Published Versus Unpublished Trials

Trial characteristics		Completed (*n* = 82)	Discontinued (*n* = 57)	Published (*n* = 72)	Unpublished (*n* = 67)	Total (*n* = 139)
Intervention	Biological	1 (1.2%)	4 (7.0%)	1 (1.4%)	4 (6.0%)	5 (3.6%)
	Device	4 (4.9%)	2 (3.5%)	5 (6.9%)	1 (1.5%)	6 (4.3%)
	Drug	33 (40.2%)	25 (43.9%)	29 (40.3%)	29 (43.3%)	58 (41.7%)
	Procedure	6 (7.3%)	2 (3.5%)	1 (1.4%)	7 (10.4%)	8 (5.8%)
	Radiation	3 (3.7%)	6 (10.5%)	3 (4.2%)	6 (9.0%)	9 (6.5%)
	Drug and procedure	4 (4.9%)	1 (1.8%)	3 (4.2%)	2 (3.0%)	5 (3.6%)
	Drug and radiation	16 (19.5%)	10 (17.5%)	18 (25.0%)	8 (11.9%)	26 (18.7%)
	Procedure and radiation	3 (3.7%)	3 (5.3%)	2 (2.8%)	4 (6.0%)	6 (4.3%)
	Multiple interventions	9 (11.0%)	3 (5.3%)	8 (11.1%)	4 (6.0%)	12 (8.6%)
	Other	3 (3.7%)	1 (1.8%)	2 (2.8%)	2 (3.0%)	4 (2.9%)
Funding	Industry	20 (24.4%)	8 (14.0%)	17 (23.6%)	11 (16.4%)	28 (20.1%)
	Industry and other^*a*^	3 (3.7%)	4 (7.0%)	3 (4.2%)	4 (6.0%)	7 (5.0%)
	NIH	2 (2.4%)	1 (1.8%)	1 (1.4%)	2 (3.0%)	3 (2.2%)
	NIH and other^*b*^	14 (17.1%)	5 (8.8%)	12 (16.7%)	7 (10.4%)	19 (13.7%)
	Other^*c*^	43 (52.4%)	39 (68.4%)	39 (54.2%)	43 (64.2%)	82 (59.0%)
Location	International	45 (54.9%)	35 (61.4%)	39 (54.2%)	41 (61.2%)	80 (57.6%)
	United States	37 (45.1%)	22 (38.6%)	33 (45.8%)	26 (38.8%)	59 (42.4%)

^*a*^Industry and other: Industry plus nonprofit organization (*n* = 3), industry plus a hospital or university, government, and nonprofit organization (*n* = 4).

^*b*^NIH and other: NIH plus nonprofit organization (*n* = 12), NIH plus a hospital or university (*n* = 7).

^*c*^Other: Hospital or university (*n* = 56), nonprofit organization (*n* = 17), nonprofit organization plus a hospital or university (*n* = 5), private (n = 1), government plus private (*n* = 1), nonprofit organization plus private (*n* = 1), government (*n* = 1).

### Trial Discontinuation

**Figure 2.**
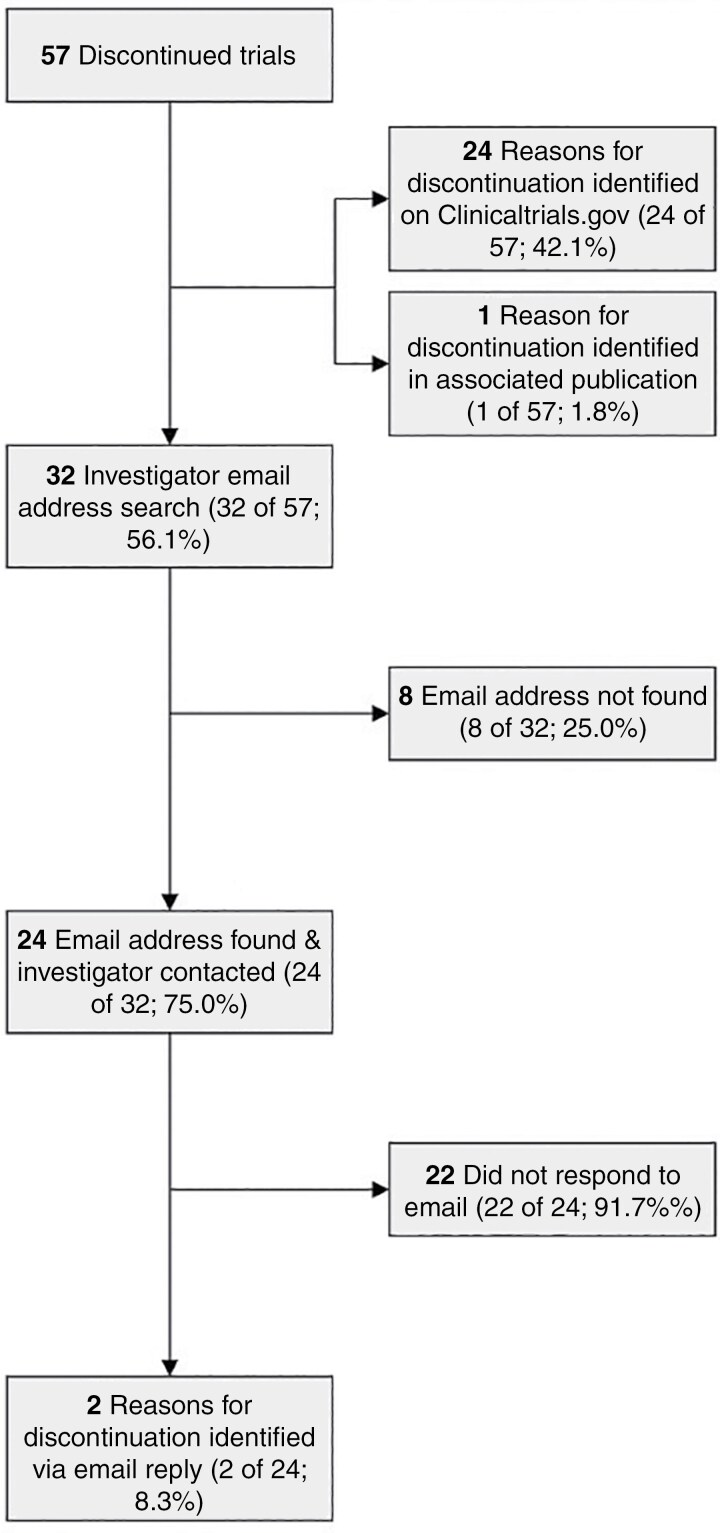
Flowchart for discontinued trials.

In our sample, 57 of the 139 (41.0%) trials were discontinued and 82 of the 139 (59.0%) were completed. Of the discontinued trials, 8 (14.0%) had a status of withdrawn, 20 (35.1%) were terminated, and 29 (50.9%) had an unknown status. There were 7 (12.3%) discontinued combined phases 2 and 3 trials, 38 (66.7%) discontinued phase 3 trials, and 12 (21.1%) discontinued phase 4 trials. A reason for discontinuation was reported on Clinicaltrials.gov for 24 of the 57 trials (42.1%), while the remaining 33 trials (57.9%) provided no reason. For 1 of these 33 trials (3.0%), an associated publication was identified that described the reason for discontinuation. An email address for the corresponding author was located for 24 of the remaining 32 trials (75.0%) and after the authors were contacted, 2 of the 24 (8.3%) responded with a resultant reason for discontinuation identified (Figure 2). Contact information for other investigators on the trial listing was found for 13 of the 22 (59.1%) trials with no reason for discontinuation yet identified. After contacting these authors, no responses were received. In total, a reason for discontinuation was identified for 27 of the 57 discontinued trials (47.4%). The reasons for discontinuation that were identified include slow enrollment or accrual (13 of the 27 trials [48.1%]), protocol amendments in progress (5 of the 27 trials [18.5%]), lack of funding (2 of the 27 trials [7.4%]), negative results or no effect (2 of the 27 trials [7.4%]), principal investigator left institution (2 of the 27 trials [7.4%]), slow enrollment and lack of funding (2 of the 27 trials [7.4%]), and new research priorities (1 of the 27 trials [3.7%]).

Through email correspondence with the respective author, a publication was identified for one trial that had a status of unknown on Clinicaltrials.gov and was initially considered as a discontinued trial in our sample. The starting dates for discontinued studies ranged from November 1996 to December 2021. Of the 57 discontinued trials, 21 (36.8%) had a starting date between 2012 and 2016.

### Publication of Completed Trials

**Figure 3.**
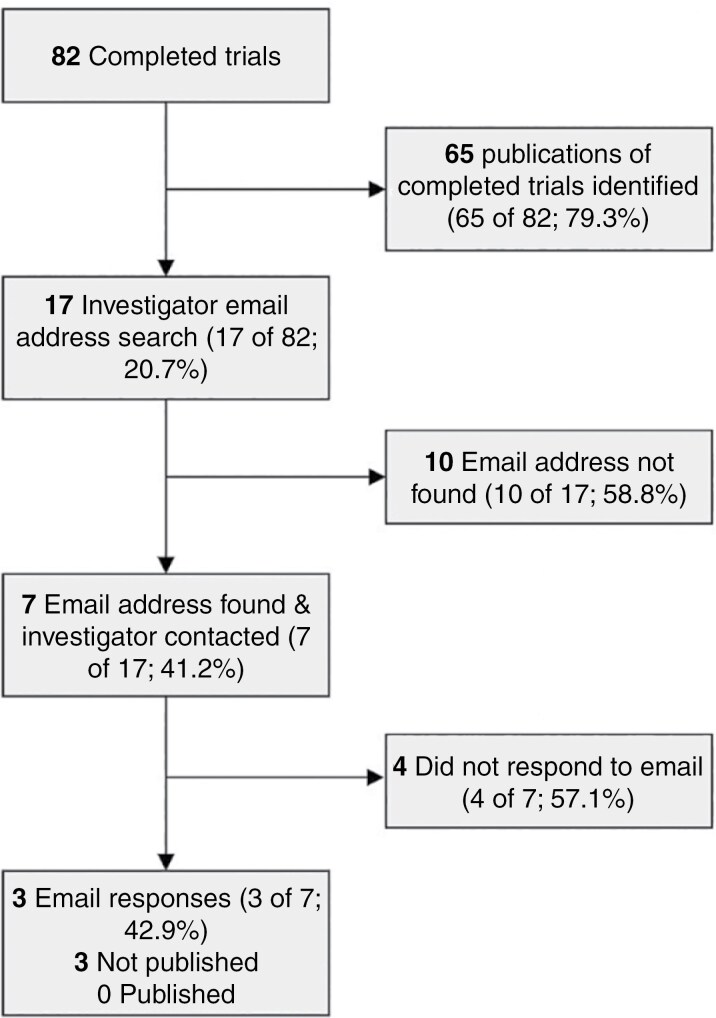
Flowchart for completed trials.

Our sample included 82 (59.0%) completed clinical trials. Of these, 4 (4.9%) were combined phases 2 and 3, 62 (75.6%) were phase 3, and 16 (19.5%) were phase 4 trials. An associated publication was found for 65 of the 82 (79.2%) completed trials after reviewing Clinicaltrials.gov and performing searches of PubMed, Embase, and Google Scholar. The investigators’ contact information was located for 7 of the remaining 17 trials (41.2%), and after sending emails we received a response for 3 out of 7 trials (42.9%). Email responses confirmed that each of the 3 trials was not published (Figure 3). No alternate investigator contact information could be located for the 4 trials for which no email response was received. The reasons for nonpublication identified include initial submission rejection and no plans for resubmission (2 of the 3) and sponsor/funding difficulties (1 of the 3), though the corresponding author for this trial also reported having a publication in preparation or under review. In addition, email correspondence with investigators identified one trial that had a status of completed on Clinicaltrials.gov but was discontinued.

### Overall Publication Status of Completed and Discontinued Studies

In our final analysis of 139 RCTs, 72 (51.8%) trials were found to be published in a peer-reviewed journal, and 67 (48.2%) were unpublished. Published trials included 3 combined phases 2 and 3 trials (4.2%), 56 phase 3 trials (77.8%), and 13 phase 4 trials (18.0%). Of the 57 discontinued clinical trials, 7 (12.3%) had a resulting publication. Our sample of 82 completed trials included 65 that were published (79.2%) and 17 that were not published (20.7%). Of the unpublished trials, 26 of the 67 (38.8%) were conducted in the United States and 41 of the 67 (61.2%) were conducted internationally. Twenty-two of the fifty-seven (38.6%) discontinued trials were conducted in the United States and the remaining 35 (61.4%) were conducted internationally. Additional trial characteristics by completion and publication status are shown in Table 1.

Clinical trials funded by sources other than industry or the NIH (which, in our sample, included nonprofit organizations, hospitals or universities, government agencies, private investors, and combinations of 2 or more of these sources) were more likely to be discontinued (adjusted odds ratio 4.24, 95% confidence interval 1.31–13.78). Trials investigating medical devices (0.04, 0.00–0.83), procedures (0.02, 0.00–0.47), pharmacological agents and procedures (0.02, 0.00–0.60), pharmacological agents and radiation (0.07, 0.01–0.93), or a combination of 3 or more interventions (0.03, 0.00–0.59) were less likely to be prematurely discontinued. Trials investigating medical devices (0.03, 0.00–0.73) and pharmacological agents and radiation (0.06, 0.00–0.88) were also less likely to be unpublished. No additional associations between intervention type, funding source, location, discontinued or unpublished clinical trials were identified with univariate or multivariate logistic regression (Table 2).

**Table 2.** Logistic Regression of Trial Discontinuation and Nonpublication^*a*^

Trial characteristics		Discontinued trials (*n* = 57)			Unpublished trials (*n* = 67)		
		No. (%)	Odds ratio (95% C.I.)		No. (%)	Odds ratio (95% C.I.)	
			Adjusted	Unadjusted		Adjusted	Unadjusted
Intervention	Biological	4 (7.0%)	1 (reference)	1 (reference)	4 (6.0%)	1 (reference)	1 (reference)
	Device	2 (3.5%)	0.0 (0.0–0.8)	0.1 (0.0–2.0)	1 (1.5%)	0.0 (0.0–0.7)	0.1 (0.0–1.1)
	Drug	25 (43.9%)	0.1 (0.0–1.0)	0.2 (0.0–1.8)	29 (43.3%)	0.1 (0.0–1.8)	0.3 (0.0–2.4)
	Drug and procedure	1 (1.8%)	0.0 (0.0–0.6)	0.1 (0.0–1.4)	2 (3.0%)	0.1 (0.0–2.1)	0.2 (0.0–2.8)
	Drug and radiation	10 (17.5%)	0.1 (0.0–0.9)	0.2 (0.0–1.6)	8 (11.9%)	0.1 (0.0–0.9)	0.1 (0.0–1.2)
	Multiple interventions	3 (5.3%)	0.0 (0.0–0.6)	0.1 (0.0–1.1)	4 (6.0%)	0.1 (0.0–1.2)	0.1 (0.0–1.5)
	Other	1 (1.8%)	0.0 (0.0–1.1)	0.1 (0.0–1.9)	2 (3.0%)	0.1 (0.0–3.6)	0.3 (0.0–4.7)
	Procedure	2 (3.5%)	0.0 (0.0–0.5)	0.1 (0.0–1.3)	7 (10.4%)	0.8 (0.0–21.2)	1.8 (0.1–36.3)
	Procedure and radiation	3 (5.3%)	0.1 (0.0–2.1)	0.3 (0.0–3.8)	4 (6.0%)	0.3 (0.0–6.0)	0.5 (0.0–8.0)
	Radiation	6 (10.5%)	0.1 (0.0–2.7)	0.5 (0.1–6.7)	6 (9.0%)	0.2 (0.0–4.3)	0.5 (0.0–6.7)
Funding	Industry	8 (14.0%)	1 (reference)	1 (reference)	11 (16.4%)	1 (reference)	1 (reference)
	Industry and other^*b*^	4 (7.0%)	1.4 (0.2–10.1)	2.5 (0.5–12.5)	4 (6.0%)	0.8 (0.1–5.3)	1.9 (0.7–5.4)
	NIH	1 (1.8%)	1.4 (0.1–20.7)	1.3(0.1–15.8)	2 (3.0%)	2.2 (0.1–36.1)	1.1 (0.3–3.7)
	NIH and other^*c*^	5 (8.8%)	0.7 (0.2–3.3)	0.9 (0.2–3.3)	7 (10.4%)	0.9 (0.2–3.6)	1.7 (0.3–9.1)
	Other^*d*^	39 (68.4%)	4.2 (1.3–13.8)	2.3 (0.9–5.9)	43 (64.2%)	1.8 (0.6–5.4)	3.4 (0.3–45.0)
Location	International	35 (61.4%)	1 (reference)	1 (reference)	41 (61.2%)	1 (reference)	1 (reference)
	United States	22 (38.6%)	2.3 (0.8–6.3)	0.8 (0.4–1.5)	26 (38.8%)	1.3 (0.5–3.3)	1.3 (0.7–2.6)

^*a*^Logistic regression adjusted for intervention, funding source, and location of trial conductance.

^*b*^Industry and other: industry plus a hospital or university, government, and nonprofit organization (*n* = 8).

^*c*^NIH and other: NIH plus nonprofit organization (*n* = 3), NIH plus a hospital or university (*n* = 9).

^*d*^Other: Hospital or university (*n* = 57), nonprofit organization (*n* = 15), nonprofit organization plus a hospital or university (*n* = 6), private (*n* = 2), nonprofit organization plus private (*n* = 2).

## Discussion

Our study revealed that almost one-half of neurooncological RCTs were discontinued. This rate of discontinuation is remarkably higher than what has been demonstrated in similar studies of other conditions including chronic pain, osteoarthritis, urological conditions, and alcohol use disorder as well as in studies of RCTs as a whole.^20,22–25^ Clinical trials require significant financial, physical, and human investments and when prematurely halted, impose financial and scientific burdens while failing to achieve their scientific goals.^26^ This is of particular concern in clinical trials of brain and other nervous system tumors considering the high mortality and poor prognosis that is often associated with these diagnoses.

We found that studies investigating biological agents had the highest rate of discontinuation, with approximately 8 in 10 associated trials being discontinued. Several challenges in the development of biological therapies have been previously identified, including the need for parenteral administration, complex manufacturing, safety concerns related to immunogenic potential, and the often-prolonged time required to assess toxicology.^27^ These factors likely contribute to increased trial discontinuation due to additional difficulties with patient enrollment and retention as well as increased costs and sponsor or funding issues. Additionally, our analysis identified a significant association between funding source and trial discontinuation, with trials funded by sources other than industry or the NIH (including hospitals or universities, nonprofit organizations, private investors, and government funding sources) being more likely to be discontinued. These findings suggest the need for targeted interventions to support ongoing neurooncological research, particularly for trials funded by nontraditional sources.

In our analysis of the reasons for discontinuation of neurooncological clinical trials, over one-half of RCTs were discontinued for unknown reasons. A low response rate from trial investigators likely contributes to this finding and warrants further investigation, particularly because of its inconsistency with similar research in other medical specialties. For example, a 2022 study of pediatric clinical trials identified a reason for trial discontinuation for 84.1% of their sample.^28^ Similarly, in a 2018 study of osteoarthritis clinical trials, a reason for discontinuation was found for 29 of the 30 included trials.^20^ In comparison, a reason for discontinuation was identified for 47.4% of trials in our study with a response rate of approximately 8%.

In addition to the prevalence of discontinuation in neurooncological clinical trials, our findings suggest the need for greater transparency and consistency in reporting the reasons for trial discontinuation in public registries like Clinicaltrials.gov. Among studies with identified reasons for discontinuation, slow enrollment or accrual was the most reported cause. This is consistent with a 2022 study of glioblastoma-related clinical trials, which reported participant accrual difficulties as the leading cause of early trial termination.^29^ Though recruitment of participants is regarded as one of the more common areas of difficulty in conducting RCTs, previous studies suggest that most reasons for recruitment failure were preventable.^30,31^ To reduce research waste, it’s important to address discontinuation resulting from avoidable factors like inadequate recruitment, flawed study design, financial concerns, and evolving priorities of the investigators and/or sponsors. This therefore highlights an opportunity to mitigate research insufficiency in neurooncological clinical trials.

Furthermore, nearly one-half of the clinical trials in our sample were unpublished. These included about quarter of the completed trials and over three-fourths of the discontinued trials. Our finding that completed trials were more likely to reach publication than discontinued trials is consistent with previous studies.^18,31^ Results from discontinued trials as well as missing data are important in clinical research, and failure to share this information with the scientific community compounds the wasting of scarce public resources that occurs with trial discontinuation. In addition, if the scientific community is not informed about the obstacles that lead to the termination of randomized controlled trials, there is a risk that the same errors will be made again. The findings from these halted and consequently, underpowered RCTs could be inconclusive. Nonetheless, these outcomes are still valuable, offering crucial preliminary data for subsequent studies and potentially contributing to systematic reviews and meta-analyses.^32^

Taking steps to reduce the discontinuation and nonpublication of clinical trials is imperative to reduce research waste, further our knowledge, and ultimately improve outcomes for patients with brain and other nervous system tumors. Given that many trials in our sample were halted due to issues with recruiting and retaining participants, we argue that those conducting clinical trials should prioritize establishing measures to prevent trial termination due to avoidable circumstances. A 2013 study by Treweek and colleagues investigated methods for improving recruitment in RCTs and indicated several interventions that appear to improve participant recruitment, such as telephone reminders to non-responders and establishing procedures for potential participants to opt out of further contact with the research team if they do not want to participate in a trial.^33^ Implementation of strategies such as these in neurooncological clinical trials may improve the recruitment of participants and reduce discontinuation of trials in the future. Previous studies indicate that the publication of clinical trials with negative results takes longer and occurs at a lower rate than other trials.^34,35^ This issue is not limited to clinical trials related to nervous system tumors and occurs throughout all medical specialties. The inclusion of a section for negative results in scientific journals has been done in other specialties and should be considered in all neurooncological-related journals to reduce publication bias and prompt authors to submit their findings, irrespective of the nature or perceived significance.^36^

### Limitations

We acknowledge several factors that may have affected the results of our study and therefore recommend that readers interpret our findings accordingly. First, though ClinicalTrials.gov is the largest primary registry in the World Health Organization International Clinical Trials Registry Platform (ICTRP), it does not include all clinical trials worldwide.^37^ Additionally, although our search results included those found based on the automated term-mapping capability of ClinicalTrials.gov, we used selected keywords to locate trials. For these reasons, it is possible that not all relevant clinical trials were captured in our study. Though we followed all standard measures to determine the publication status for trials in our sample, there may have been publications that were not found. Furthermore, because the information on Clinicaltrials.gov is largely provided by investigators and sponsors, we were not able to verify the accuracy of the trial data. Lastly, our study may not be generalizable to all clinical trial types as we primarily included phases 3 and 4 trials.

## Conclusions

Our study demonstrated that discontinuation of neurooncological clinical trials was common and often occurred for unknown reasons. We also found that trials were frequently unpublished, particularly those that were discontinued. Discontinuation and nonpublication of trial findings hinder our ability to improve care for patients with brain and other nervous system tumors. Until the issues described in our study are addressed, they will continue to limit the advancement of the field and contribute to the wasting of research resources.

## Data Availability

The datasets generated during and/or analyzed during the current study are available from the corresponding author on reasonable request.
